# User experience of self‐reported computerized medical history taking for acute chest pain: The Clinical Expert Operating System Chest Pain Danderyd Study

**DOI:** 10.1111/hex.13612

**Published:** 2022-09-23

**Authors:** Kay Sundberg, Athena Adeli, Helge Brandberg, Jonas Spaak, Sabine Koch, Carl J. Sundberg, David Zakim, Thomas Kahan, Kaisa Fritzell

**Affiliations:** ^1^ Department of Neurobiology, Care Sciences and Society, Division of Nursing Karolinska Institutet Stockholm Sweden; ^2^ Department of Learning, Informatics, Management and Ethics, Medical Management Centre, and Health Informatics Centre Karolinska Institutet Stockholm Sweden; ^3^ Department of Clinical Sciences, Division of Cardiovascular Medicine, Danderyd Hospital Karolinska Institutet Stockholm Sweden; ^4^ Department of Physiology & Pharmacology Karolinska Institutet Stockholm Sweden; ^5^ Cancer Theme, Reception Hereditary Cancer Karolinska University Hospital Stockholm Sweden

**Keywords:** chest pain, computerized history taking, emergency department, health informatics, medical history, user experience

## Abstract

**Background and Objective:**

Chest pain is one of the most common complaints in emergency departments (EDs). Self‐reported computerized history taking (CHT) programmes can be used for interpretation of the clinical significance of medical information coming directly from patients. The adoption of CHT in clinical practice depends on reactions and attitudes to the technology from patients and their belief that the technology will have benefits for their medical care. The study objective was to explore the user experience of the self‐reported CHT programme Clinical Expert Operating System (CLEOS) in the setting of patients visiting an ED for acute chest pain.

**Methods:**

This qualitative interview study is part of the ongoing CLEOS‐Chest Pain Danderyd Study. A subset (*n* = 84) of the larger sample who had taken part in self‐reported history taking during waiting times at the ED were contacted by telephone and *n* = 54 (64%) accepted participation. An interview guide with open‐ended questions was used and the text was analysed using directed content analysis.

**Results:**

The patients' experiences of the CLEOS programme were overall positive although some perceived it as extensive. The programme was well accepted and despite the busy environment, patients were highly motivated and deemed it helpful to make a diagnosis. Six categories of user experience emerged: The clinical context, The individual context, Time aspect, Acceptability of the programme, Usability of the programme and Perceptions of usefulness in a clinical setting.

**Conclusions:**

The programme was well accepted by most patients in the stressful environment at ED although some found it difficult to answer all the questions. Adjustments to the extent of an interview to better suit the context of the clinical use should be a future development of the programme. The findings suggest that CHT programmes can be integrated as a standard process for collecting self‐reported medical history data in the ED setting.

## Patient or Public Contribution

In the conduct of this study, patients contributed with interviews reflecting their usage of a self‐reported medical history‐taking programme.

## 1 INTRODUCTION

Quality of care for individual patients depends on having access to the care they need, and the effectiveness of the care provided.^1^ The latter requires that individual circumstances and the complexity of the individual's clinical issues are determined accurately. Primary teaching in this regard is that the information a patient can report to their physician in response to clinically informed questioning is paramount.^2^, ^3^ However, as medical history taking is based on an extraordinarily large body of empirical knowledge this becomes a knowledge‐intensive and time‐consuming task. Individual physicians cannot master the nearly infinite body of knowledge for exhaustive history taking.^4^ Patients may detect physician stress making them feel less comfortable and impacting their feelings of trust in the quality of the care delivered.^5^

The knowledge base for history taking and for interpreting the clinical significance of the information collected can be formalized as software to enable computerized history taking (CHT) directly from patients.^6^, ^7^ CHT programmes have shown to collect more complete and accurate historical data, as compared to physicians examining the same patients.^7^, ^8^, ^9^ Clinical Expert Operating System (CLEOS) is a CHT software for self‐reported medical histories entered by patients on a tablet.^10^ We currently study the implementation of CLEOS as a tool for managing patients who come to an emergency department (ED) with acute chest pain. This is one of the most common complaints in EDs, accounting for 5%–12% of the visits.^11^, ^12^, ^13^

The first results from the CLEOS‐Chest Pain Danderyd Study (CLEOS‐CPDS) show that most patients can interact effectively with a CHT programme to provide sufficient data for risk stratification of acute chest pain.^14^ That report did not provide any data on patients' experiences of using such programmes which is also important to collect. The adoption of CHT in clinical practice depends, among other issues, on positive responses to the technology from patients and the belief by patients that the technology will have benefits for their medical care. When evaluating a CHT programme, user experience is a term that covers meaningful and valuable aspects of the interaction between humans and computers.^15^, ^16^ The user experience will be influenced by predispositions, expectations, motivations, the environment within which the interaction takes place and the usability and functionality of the system. So far, knowledge is scarce as to how patients react to using CHT programmes and how they would like to contribute to the further development and deployment of such programmes.^17^, ^18^ Thus, the objective of this study was to explore the user experience of the self‐reported CHT programme CLEOS in the setting of patients visiting an ED for acute chest pain.

## 2 MATERIALS AND METHODS

### 2.1 Design

This qualitative interview study is part of the CLEOS‐CPDS,^10^ which has been approved by the Stockholm Regional Ethics Committee (reference number 2015/1955‐1) and is registered at https://www.clinicaltrials.gov.

**Figure 1:**
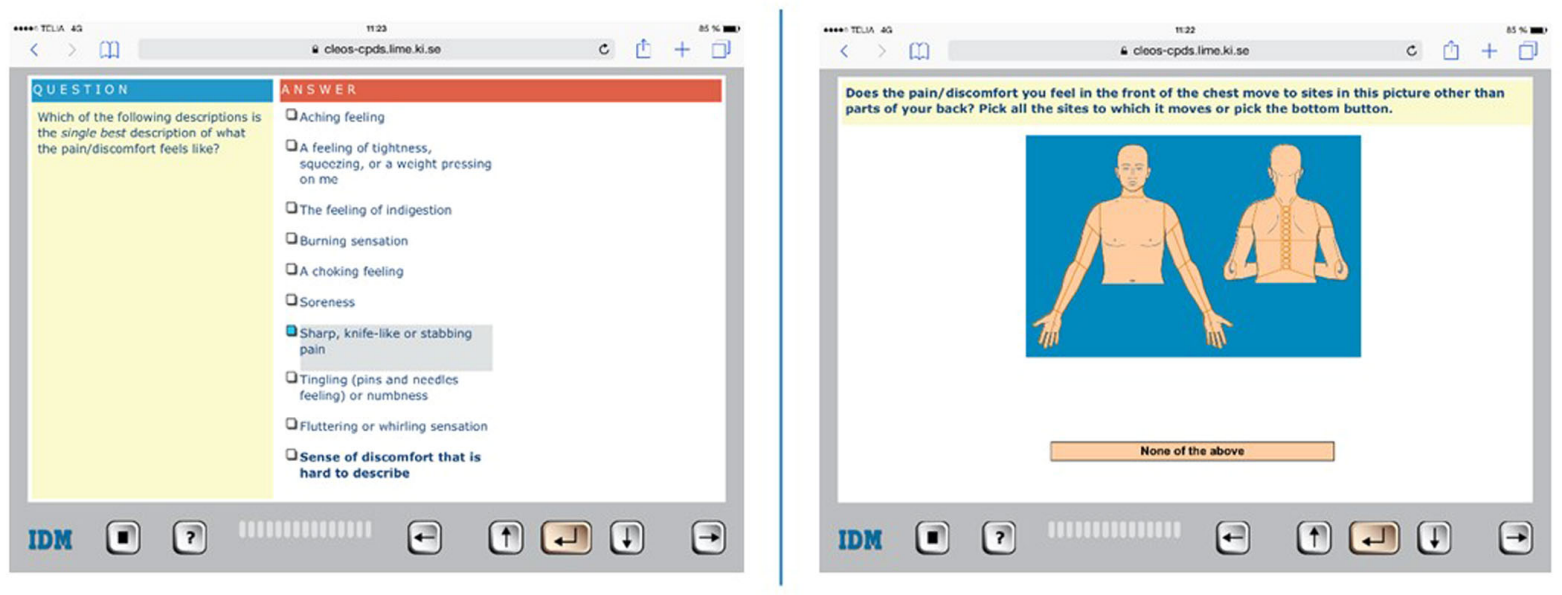
Example of questions in Clinical Expert Operating System (CLEOS) on the tablet

The CLEOS‐CPDS study has been running at the cardiology ED at Danderyd University Hospital (Stockholm, Sweden) since October 2017 and aims to recruit a total of 2000 patients consecutively until 2023. Inclusion criteria include women and men aged 18 years and older with chest pain who are attending the cardiology ED, triaged to the waiting room before medical evaluation, and fluency in Swedish. Excluded are critically ill patients and those who due to confusion or agitation have the inability to carry out a CHT interview. The patient's self‐reported history taking by the CLEOS programme is performed during waiting times. By clicking on an individually tailored question, the patient answers questions regarding present illness, past medical history, lifestyle risks, and family history (Figure 1). Depending on the answer, the programme determines dynamically the next most appropriate question. In all, CLEOS can collect >40,000 data elements with >17,000 decision nodes. However, the patients are only asked a fraction of these as the interview is unique for each patient. The median time to collect data to assess chest pain and subsequent cardiovascular risk was 23 min and to complete the full interview 64 min.^14^

### 2.2 Study sample

While obtaining informed consent in the CLEOS‐CPDS study the patients were informed that they might be asked later to take part in an evaluation of their perceptions of using the programme. Approximately, 800 patients had been included in the CLEOS‐CPDS study at the time of the present interview study and a convenience sample of all patients (*n* = 84), who had taken part during a 2‐month period from July to August 2019, were eligible to be included. These patients were contacted by phone within 3–11 weeks after their ED visit by the three authors who conducted the interviews. A total of 54 (64%) patients took part; 5 patients declined to participate; 27 could not be reached. The mean (range) age of the patients was 63 (34–85) years, and 20 (37%) were women.

### 2.3 Interviews

When contacted, patients were asked whether they were willing to take part in an interview over the phone straight away or whether they preferred another, more convenient time. Patients not reached at the first call were recontacted twice and all interviews were performed over a 4‐week period. A structured interview guide with open‐ended questions was developed informed by the concept of user experience; predispositions, expectations, motivations, the environment, usability and functionality.^15^, ^16^ The guide included four key questions and several probe questions (Table 1). The interviews lasted between 5 and 15 min and, were thoroughly documented in a template included in the interview guide. When the interviewer did not understand a patient's answer, the documented text was represented to the patient for eventual clarification.

**Table 1:** Interview guide

Key questions	Probe questions
How was your experience of responding to the questions regarding your symptoms and health on an iPad?	How did it feel given your current health situation? What was it like considering the environment? Did you feel involved in any way? What was it like timewise?
How did you experience the questions?	Did you find them relevant? Any missing questions? The number of questions?
How did you experience the technology, when using the iPad?	Did it work? Was it self‐instructive? Did you need help? What was the interface/layout like? Any suggestions for improvement?
Do you think that CLEOS could be useful or have a role in health care?	

Abbreviation: CLEOS, Clinical Expert Operating System.

### 2.4 Analysis

The interviews were merged into one unit of analysis, as text, and a coding scheme was created using the four questions in the interview guide. The text was analysed by the same three authors who performed the interviews using directed content analysis.^19^ Specific words or text content related to the questions in the interview guide were identified and coded. The codes then were classified into mutually exclusive subcategories based on how the different codes were related. Finally, the subcategories were classified into main categories that reflected the central content (Table 2). The classifications into subcategories and main categories were repeatedly discussed among the three authors during the analysis process and were subsequently verified by all co‐authors until a consensus was reached.

**Table 2:** An example of the analysis process of the experience of responding to the questions in CLEOS

Codes	Subcategories	Main categories
Tempting since it was about my health Comforting to respond to the questions A good way to engage the patient Good to fill in (response) to give basic data Good if you can give information beforehand	Felt good and worthwhile	The individual context
Don't remember, was dazed and scared Didn't manage to respond to all (questions) Didn't feel well, difficult to answer Needed some assistance because I didn't feel so well	Not in shape	

Abbreviation: CLEOS, Clinical Expert Operating System.

## 3 RESULTS

Six categories emerged: The clinical context, The individual context, Time aspect, Acceptability of the programme, Usability of the programme and Perceptions of usefulness in a clinical setting (Table 3).

**Table 3:** Categories, examples of quotes and main results

Main categories	Subcategories	Example of quotes	Main result
The clinical context	The impact of the environment	A bit busy environment, but it worked out.	A majority thought CLEOS had worked well in the ED and was something positive to do while waiting.
	Good pastime while waiting	It felt good to answer the questions, something was happening, and it was not just a long wait.	
The individual context	Felt good and worthwhile	It felt good, comforting, interesting, and fun.	Responding to questions about own health was perceived as positive although some did not have the strength to respond to all the questions.
	Not in shape	I don't remember … I was dazed and scared.	
Time aspects	Enough time	It took a while to answer them all, but I managed.	Some patients thought there had been enough time to answer the questions but most experienced insufficient time to complete the interview within the available period.
	Shortage of time	I didn't have time to finish, I would have wanted to answer all questions since it was a mapping of me possibly leading to something.	
Acceptability of programme	Various views of questions	It felt like one could give the wrong answers. I noticed that the program started to think I had had a heart attack, but I knew it had to do with my stomach.	Most patients had a positive view of CHT. Overall, the questions were perceived as highly relevant. Sometimes it was difficult to select answers to questions perceived as less relevant to their clinical problem. Many patients felt they were asked too many questions.
	A lot of questions	Should be shortened; it's better with more precise questions so that you don't get tired while answering.	
Usability of the programme	User‐friendly	worked completely, very self‐instructive	A majority thought CLEOS was relatively easy to use and technically safe. The visuals in the programme were perceived as helpful. Some patients made suggestions for improving the programme layout.
	Conditions for performing efficiently	It was a little slow when pointing at the different figures, unprecise and inert for instance it was difficult to point on the little toe as it hit the whole foot instead.	
Perceptions of usefulness in a clinical setting	Diagnosis facilitator	A good tool for the doctors.	Patients felt it was useful to contribute information about their health as a complement to the information otherwise available to the physician for diagnosis. Patients also believed that technology like CHT could facilitate communication in health care.
	Technical development in health care	I think that it (CHT) has a place in health care, it's good that the patient may have their say	

Abbreviations: CHT, computerized history taking; CLEOS, Clinical Expert Operating System.

### 3.1 The clinical context

A majority of patients thought CLEOS had worked well in the ED and was something positive to do while waiting.

#### 3.1.1 The impact of the environment

Despite sitting in the waiting room or lying on a stretcher in the ED, most patients found the environment acceptable when answering the questions posed by CLEOS. Only occasionally, the ED was found to be loud, messy and unsuitable for answering the sometimes demanding questions. Being interrupted for an examination or blood test was considered disturbing by some but was rarely experienced as a problem.

#### 3.1.2 Good pastime while waiting

For most of the patients, answering the questions was something that could occupy their minds and help time pass while waiting in the ED. It was appreciated to have something to focus on while waiting and, answering questions posed by CLEOS was experienced as helpful to turn off the surroundings.

### 3.2 The individual context

Responding to questions about their health was perceived as positive although some did not have the strength to respond to all the questions.

#### 3.2.1 Felt good and worthwhile

Using CLEOS was viewed as a positive experience by many patients. Most stated that it felt good to respond to the questions concerning their own health and good to contribute to research. Further, it was found positive to provide basic data about their health before seeing the doctor. Some expressed that the use of CLEOS was a good way of engaging and involving the patients in their own care.

#### 3.2.2 Not in shape

Some patients expressed they were stressed, worried, scared, and bemused when coming to the ED. They had felt very ill at the time and did not remember much from this occasion. Some said that they did not have the strength at the time to respond to all the questions.

### 3.3 Time aspects

Some patients thought there had been enough time to answer the questions, but most experienced insufficient time to complete the interview within the available period.

#### 3.3.1 Enough time

There were patients who expressed that there had been plenty of time to answer the questions in the CLEOS interview and that they had managed to answer them all quite quickly. There were also some patients who mentioned that it had taken a long time to complete the interview, but they had not considered the time aspect as negative.

#### 3.3.2 Shortage of time

Most patients thought that it took a long time to answer the questions. It was stated often that there had been insufficient time to manage all questions and that they had been interrupted by examinations or even discharged before they had the opportunity to complete the interview.

### 3.4 Acceptability of programme

Most patients had a positive view of CHT. Overall, the questions were perceived as highly relevant. Sometimes, however, it was difficult to select answers to questions perceived as less relevant to their clinical problem. Many patients felt they were asked too many questions.

#### 3.4.1 Various views of questions

The questions were perceived as general, but also specific in the sense that they supplemented each other with follow‐up questions. Most perceived all questions as smart and relevant to the chief complaint regarding their visit. Some did not recall what the questions were about, but they remembered them as being relevant at the time of answering them. Nobody perceived the questions as intimidating but sometimes a bit trivial and intimate when concerning bowel movements and urinary function. The questions were often considered highly relevant at first, but subsequently, they appeared less relevant and thus more difficult to answer with the most appropriate response alternative. Then, when the relevance decreased, the least wrong answer had to be selected to be able to move forward in the programme. If the chosen answer was out of the scope, the consequence was a perception of a series of irrelevant questions or that the programme got stuck on the wrong aspect. Irrelevant questions created frustration, and, in some cases, the informants decided to stop answering altogether.

#### 3.4.2 A lot of questions

The majority expressed that there had been too many questions, giving a notion that they would never end. Some expressed this as frustrating, but some thought it was acceptable as the questions felt important. Many of those who thought there was an abundance of questions also found it difficult to understand the connections between the questions. There were statements describing the questions as repetitive, very similar to each other, and tiresome to answer.

### 3.5 Usability of programme

A majority thought CLEOS was relatively easy to use and technically safe. The visuals in the programme were perceived as helpful. Some patients made suggestions for improving the programme page layout.

#### 3.5.1 User‐friendly

The general perception was that it had been easy to use the CLEOS programme on a tablet. Some patients with less experience with tablets thought they quickly got used to it, but some patients stated they needed help to get started. For most, CLEOS had felt technically safe to use but some who had accidentally turned it off required some assistance to restart the programme. Also, there were instances in which the programme stopped working and help was required to reboot the tablet. Occasionally the programme had been very slow and lagging when moving from one page to the next.

#### 3.5.2 Conditions for performing efficiently

The layout was mostly described as simple and self‐instructive, and it was easy to move forward in the programme as in a regular questionnaire. The visuals in the programme were perceived as helpful, that is, the figure of the torso where they could point out where they experienced pain. Some found some features less easy to use. A common disturbing problem was the need to scroll down a page to find the button to move to the next question. Some patients were bothered by the absence of information on their progress in completing the interview. There were some suggestions for improving page layout by moving the scroll‐down button to the top of a question page. Some patients also suggested that the programme pages should indicate progress to completing an interview.

### 3.6 Perceptions of usefulness in a clinical setting

Patients felt it was useful to contribute information about their health as a complement to the information otherwise available to the physician for diagnosis. Patients also believed that technology like CHT could facilitate communication in health care.

#### 3.6.1 Diagnosis facilitator

There were positive perceptions about CLEOS providing information directly from the patient as a complement for the physician when making a diagnosis. Some thought that it might be difficult for a physician to decide on a diagnosis and that CLEOS could help. It was also perceived to be good to use CLEOS when the physician was too busy to question the patient and some thought that it might speed up the diagnostic process.

#### 3.6.2 Technical development in health care

Many could see the benefits of technical devices in health care. They thought that technical development is good and needed and that CLEOS might facilitate communication in health care. Only one patient expressed a negative view and thought that it would be bad if the technology takes control of the care of patients.

## 4 DISCUSSION

This study shows that most patients attending the ED for chest pain had a positive experience and rarely encountered problems when using the self‐reported CHT programme. The questions posed by the computerized interview were often considered to be concrete and relevant. Overall, responding to questions related to own health was perceived helpful for diagnostics and there were several positive statements regarding the role of a programme such as CLEOS in health care. Many patients thought, however, that there were too many questions to answer and insufficient time to manage them all.

The ED setting is often chaotic with limited space for privacy. Despite this, most patients in this study thought it was doable to engage in the CLEOS programme and that it was a positive distraction from the busy environment. However, a few patients, judged fit enough by staff to stay in the waiting room felt they were limited by their symptoms and lacked the strength to answer all the questions. The patient's predisposition is an influencing factor for the overall user experience^15^ and undoubtedly something to take into consideration when introducing technology into health care. It is important to note that the CLEOS programme is a complement to usual care. No risk to patients occurred in this study when the CHT interview was incomplete. The study protocol did not include a provision to complete the CHT interviews after hospitalization, but there are no technical barriers to implementing this scheme to facilitate the completion of interviews.

Motivation and feeling meaningfulness have been identified as parts of the user's internal state, important to acknowledge in the context of user experience.^15^, ^16^ There were frequent comments on the abundance of questions to answer, and a few participants lost their motivation and ended the interview prematurely. Nevertheless, most kept on self‐reporting until they were interrupted for an examination or laboratory sampling, although they thought there were too many questions and it had taken a long time to answer them. The previous study reporting from CLEOS‐CPDS noted that the proportion of patients who continued with the programme interview decreased the longer it went on.^14^ However, the most important factors for assessing chest pain were asked in the very first part of the interview which gave sufficient data to calculate well‐established risk scores for acute chest pain patients.

Motivation could most probably be connected to the perceived high relevance of the questions in relation to health concerns. It was found encouraging to share unfiltered health data before seeing the doctor thinking that CLEOS was a ‘diagnosis facilitator’. These results confirm that the patients consider their personal experience of illness and medical history important information for an accurate diagnosis.^3^

The self‐reported medical history system is programmed to take a thorough, but exhaustive history. CLEOS is a complex programme structured so that the next most appropriate question is determined by the responses to previous questions. In addition, CLEOS requires patients to select answers from preset menus of possible answers. This structure seemed to work well for most patients although some testified differently. Some had been unable to answer truthfully because the menu of possible answers did not include an alternative that was relevant for them. Maybe, it was difficult to be precise about the symptoms when this was a new experience as opposed to those who could recognize the symptoms from a previous event. For example, the question that was perceived irrelevant by a patient as he thought it concerned the heart rather than the stomachache he experienced. This is an issue to be aware of as the validity of the programme could be compromised if the user decides to come up with just any answer to be able to move forward in the programme.

Usability and functionality are additional parts of the importance of a system and should be evaluated.^15^, ^16^ The CLEOS programme on the tablet was described as user‐friendly, in line with the findings of two earlier studies at an ED where most patients felt that those CHT programmes were very easy to use.^17^, ^18^ Many questions in the CLEOS programme display images to indicate where, for example, pain is located. These were mostly appraised as positive, but there were also a few suggestions for improvements of functionality that might be taken into consideration for the future development of the programme.

Notably, almost everyone supported technical development in health care and considered that a CHT programme could help them to communicate better with their doctor. Almost all patients felt motivated to use CHT again. This is in line with the results of Benaroia et al.^18^ and Arora et al.^17^ in the emergency department who reported that over 90% of patients were strongly positive and willing to use a CHT programme again especially because it helped them organize their thoughts before seeing the physician. Some patients also expressed that using CLEOS was a good way of engaging them in their care. Patient participation is an established concept that has been strengthened through laws and regulations^20^ importantly pointing at patients' rights to influence and engage in their care in dialogue with healthcare professionals.^21^ Many patients also found that participation in the CLEOS programme was important if it was helpful to research. People are strongly motivated to participate in research studies when they feel that the knowledge gained is being shared back in a way that can lead to individual and societal benefits.^22^ The patient's involvement in testing CLEOS is an important effort to enable the development of the programme in the future. When both patients and healthcare professionals gain from the healthcare technology and consider it supportive, the likelihood of successful implementation, use, and user engagement is increased.^23^

The study represents a small sample in the ED setting and a qualitative approach was considered to give a deeper understanding of patients' reactions to and perceptions of a CHT programme. When conducting telephone interviews there are several reasons for declining participation, which may cause a selection bias. However, the relatively high inclusion number, given an interview study, is a strength and provided a richness of data for analysis. The CLEOS‐CPDS study adopts a consecutive sampling strategy over several years. The present sample included patients interacting with CLEOS only during a short segment of the overall period of patient enrolment. Moreover, the patients that accepted to participate in telephone interviews may have been those with a very positive attitude to the programme or to taking part in a study. We did not stratify the sample to make a greater variation in different background factors among the participants but still, the convenient sampling generated a representative age range and sexes of patients enrolled in the overall programme. Even if the objective was not to compare between groups nor to generalize the results, the variation generated a heterogeneous group valuable for the analysis. Our patient interviews by phone were not recorded; but given the structured interview guide made the interviews, easy to substantiate. If in doubt whether the notes that were taken during the interview were correct, the authors went over the answers again with the participants checking for validity. Adhering to the coding scheme during the analysis facilitated the organization and the trustworthiness of the categories. Our data may be subject to recall bias because our interviews were not performed immediately after the completion of CLEOS interviews. In addition, the health condition of the patients at the time of visiting the ED could have had an impact on how they remembered the CLEOS interview. We did not actually ask about previous experience of using a tablet which could have been useful for understanding the results. However, the results reveal statements when patients were not so familiar with using tablets and how they handled the situation.

## 5 CONCLUSION

The patient's experience of the CLEOS programme at a cardiology ED reflects an overall positive attitude in terms of programme properties and the usage context. The results show that the patient can be an active and willing participant in an electronic history‐taking process. The programme was well accepted by patients in a stressful situation. Despite the busy environment, CLEOS was found user‐friendly, and patients were highly motivated as they perceived it helpful to obtain a correct diagnosis. The programme can be perceived as extensive, especially by patients who feel seriously compromised by the acute distress of their health and by those who find it difficult to be precise about their symptoms. Future development of the programme should include adjustments to the extent of an interview to better suit the context of the clinical use. Taking this into consideration, the results from this study suggest nevertheless, that CHT programmes can be integrated as a standard process for collecting self‐reported medical history data in the ED setting.

## AUTHOR CONTRIBUTIONS

**Kay Sundberg**: Conceptualization; acquisition of data; analysis and interpretation of data; writing–original draft. **Athena Adeli**: Conceptualization; acquisition of data; analysis and interpretation of data; writing–review & editing; approval of the final version of the manuscript. **Helge Brandberg**: Conceptualization; analysis and interpretation of data; writing–review & editing; approval of the final version of the manuscript. **Jonas Spaak**: Conceptualization; analysis and interpretation of data; writing–review & editing; approval of the final version of the manuscript. **Sabine Koch**: Conceptualization; analysis and interpretation of data; writing–review & editing; approval of the final version of the manuscript. **Carl J. Sundberg**: Conceptualization; analysis and interpretation of data; writing–review & editing; approval of the final version of the manuscript. **David Zakim**: Conceptualization; analysis and interpretation of data; writing–review & editing; approval of the final version of the manuscript. **Thomas Kahan**: Conceptualization; analysis and interpretation of data; writing–review & editing; approval of the final version of the manuscript. **Kaisa Fritzell**: Conceptualization; acquisition of data; analysis and interpretation of data; writing–review & editing; approval of the final version of the manuscript. All authors have been part of the development of the interview guide for this study, the analysis of data and writing the manuscript.

## CONFLICT OF INTEREST

The authors declare no conflict of interest.

## ETHICS STATEMENT

This qualitative interview study is part of the CLEOS‐CPDS, which has been approved by the Stockholm Regional Ethics Committee (reference number 2015/1955‐1) and is registered at https://www.clinicaltrials.gov (unique identifier: NCT03439449).

## Data Availability

The data that support the findings of this study are available from the corresponding author [K. S.], upon reasonable request.
